# Development of a Validated High-Performance Thin-Layer Chromatography (HPTLC) Analysis Protocol for Salivary Caffeine Used as a Probe Drug

**DOI:** 10.3390/molecules30193859

**Published:** 2025-09-23

**Authors:** K. M. Yasif Kayes Sikdar, Ahmed Shalan, Vincent Castejon, Carly Chambers, Samara Renae Coverley, Okhee Yoo, Md Khairul Islam, Tomislav Sostaric, Lee Yong Lim, Philip Burcham, Cornelia Locher

**Affiliations:** 1 Department of Pharmacy and Centre for Optimisation of Medicines, School of Health and Clinical Sciences, University of Western Australia, Crawley, WA 6009, Australia; kmyasifkayes.sikdar@uwa.edu.au (K.M.Y.K.S.); ahmedshlan19@outlook.com (A.S.); vincentcastejon@outlook.com (V.C.); okhee.yoo@uwa.edu.au (O.Y.); khairul.islam@uwa.edu.au (M.K.I.); tom@chromatechscientific.com (T.S.); lee.lim@uwa.edu.au (L.Y.L.); philip.burcham@uwa.edu.au (P.B.); 2 Division of Pharmacology and Toxicology, School of Biomedical Science, University of Western Australia, Perth, WA 6009, Australia; 3 Institute for Paediatric Perioperative Excellence, University of Western Australia, Perth, WA 6009, Australia

**Keywords:** saliva, probe drug, caffeine, validation, HPTLC

## Abstract

CYP1A2 activity plays a critical role in the metabolism of drugs such as caffeine, clozapine, propranolol, and warfarin. In pharmacogenomic studies, caffeine is a probe drug of choice for CYP1A2 phenotyping. Due to the non-invasive nature of sampling, saliva is an alternative biofluid to plasma for monitoring caffeine levels. This study reports on a validated HPTLC method for quantifying salivary caffeine levels, which can support future studies on CYP1A2 phenotyping employing caffeine as a probe drug. The HPTLC method, using silica gel 60 F254 plates and acetone/toluene/chloroform (4:3:3, *v*/*v*/*v*) as the mobile phase, has detection and quantification limits of 2.42 and 7.34 ng/band, respectively. An optimised saliva processing protocol using a 1:1 dilution with methanol was also established. Five saliva sample sets collected 0–4 h after ingestion of 100 mg caffeine were analysed using the developed and validated HPTLC method, which demonstrated that salivary caffeine concentrations peak around 1 h post ingestion and then gradually decrease over the study period. Thus, the developed HPTLC method can be used to analyse caffeine levels in saliva and to support CYP1A2 phenotyping using caffeine as a probe drug.

## 1. Introduction

**Figure 1:**
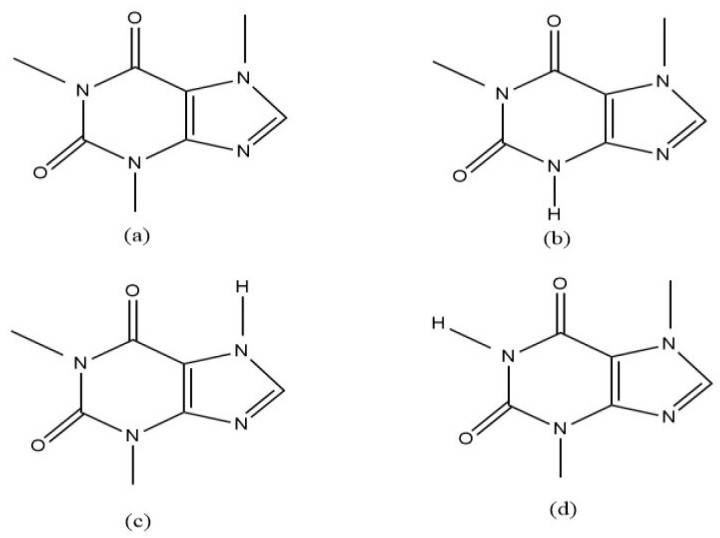
Chemical structures of caffeine (**a**) and its major metabolites paraxanthine (**b**), theophylline (**c**), and theobromine (**d**).

The metabolic capacity of cytochrome P450 (CYP450) enzymes varies substantially between individuals and influences the outcome of drug therapy [1,2]. CYP1A2, one of the most prevalent forms of the enzyme in the liver, plays a crucial role in the metabolism of drugs such as caffeine, clozapine, propranolol, warfarin, and paracetamol [2]. Pharmacogenomics, which investigates the impact of genetic makeup on a patient’s response to medication, may help predict how individuals metabolise drugs and can therefore assist in personalised drug therapy [1]. In pharmacogenomic studies, caffeine is a well-tolerated probe drug of choice for investigating CYP1A2 activity. Caffeine is predominantly metabolised by CYP1A2 (Figure 1), and its metabolism varies markedly among individuals [3,4,5,6]. In humans, caffeine is absorbed rapidly and completely after ingestion (approximately 99% within 45 min) [7,8], and plasma concentrations peak after 15 to 120 min, depending on individual CYP1A2 enzyme activity [9]. Plasma caffeine level is conventionally analysed to determine CYP1A2 phenotype, as caffeine clearance and the plasma concentration–time profile between caffeine and its main metabolite, paraxanthine, allow for distinguishing between rapid and poor metabolisers [10]. However, plasma sampling is invasive, and plasma sample processing is time-consuming [10], whereas saliva sampling offers a non-invasive alternative. Studies have also shown that salivary caffeine levels up to 4 h after ingestion correlate closely with plasma levels and therefore provide a good surrogate to determine CYP1A2 metaboliser status [11].

Bioanalytical methods for drug concentration measurements and determination of pharmacokinetic parameters commonly use high-performance liquid chromatography (HPLC) coupled to an ultraviolet (UV) spectrophotometer, fluorescence emission detector, or tandem mass spectrometric detector, as well as atomic absorption spectrometry (AAS) and induction coupled plasma mass spectrometry (ICP-MS) [12]. However, when a fast turnover and high throughput quantification are required, these methods show limited efficiency [12]. Moreover, while HPLC is commonly used to analyse saliva samples in studies investigating CYP1A2 activity using salivary caffeine as a probe drug [13,14,15], a complex and time-consuming sample extraction and residue reconstitution process makes HPLC analysis less convenient [10]. An electrochemical-based method using differential pulse voltammetry with a glassy carbon electrode was also reported to quantify paraxanthine and caffeine in saliva, but liquid–liquid extraction prior to analysis is necessary in this approach, resulting in a complex and time-consuming process [16]. Enzyme-linked immunosorbent assays (ELISA) might be a convenient alternative, but the use of commercially available test kits is prohibitively expensive for routine analysis [17].

High-performance thin-layer chromatography (HPTLC), an automated analytical tool, offers the ability to run multiple samples simultaneously and allows the detection as well as quantification of targeted molecules at nanogram levels while using only small volumes of solvents and reagents. With this, HPTLC can be considered a cost- and time-efficient analysis alternative [17,18]. Prior studies have demonstrated that HPTLC can be used to measure caffeine levels in saliva and urine at nanogram levels following ingestion [19]. However, these methods require pre-analytical extraction and overnight solvent evaporation, resulting in a complex and time-consuming analysis protocol [19]. This study, therefore, aimed to develop and validate an HPTLC method for quantifying salivary caffeine levels without complex extraction procedures and with a lower detection and quantification limit. It also aimed to demonstrate the suitability of the established method for quantifying caffeine in clinical saliva samples collected at different time points following caffeine ingestion.

## 2. Results and Discussion

### 2.1. HPTLC Method Optimisation

**Figure 2:**
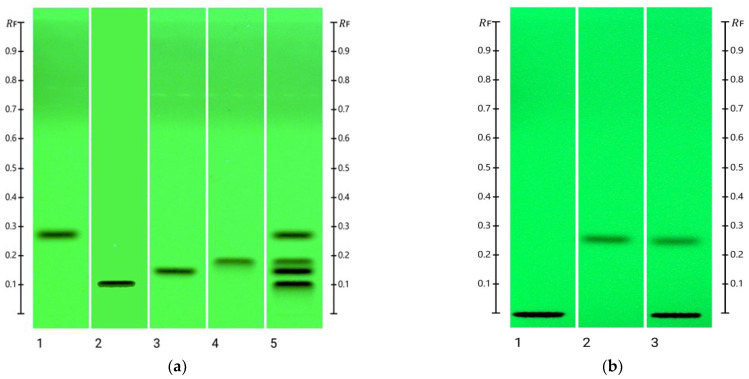
(**a**) HPTLC fingerprint at 254 nm. Track 1: Caffeine; Track 2: Paraxanthine; Track 3: Theobromine; Track 4: Theophylline; and Track 5: Mixture of all four compounds. (**b**) Image of HPTLC plate at 254 nm. Track 1: Blank saliva; Track 2: Caffeine (50 ng/band); and Track 3: Saliva spiked with caffeine (50 ng/band).

After trialling multiple solvent mixtures at different ratios (e.g., ethyl acetate, methanol, n-butanol, toluene, glacial acetic acid, acetone, chloroform, and acetonitrile), which were all used in previous studies to analyse caffeine in different matrices [19,20,21,22], acetone/toluene/chloroform (4:3:3, *v*/*v*/*v*) was selected as the mobile phase for this study, as it resulted in well-separated bands for caffeine and its three metabolites, paraxanthine, theophylline, and theobromine (Figure 2a). Using this mobile phase, which was previously used to analyse these compounds in Mate beer and soft drinks [22], caffeine produced a distinct R_F_ value of 0.25.

HPTLC fingerprints of caffeine and its metabolites were initially obtained at 254 nm and interpreted using the visionCATS 4.0 (CAMAG) software. Peak height versus concentration was found to produce the best results with high levels of reproducibility for the quantification of caffeine.

For optimal detection, caffeine’s UV–vis spectrum was also recorded using a TLC Scanner 4 (CAMAG), and its absorbance maximum (λ_max_) was determined to be at 275 nm (Figure S1), which is in line with the findings of previous studies [19]. The HPTLC method validation and subsequent caffeine quantification of all clinical samples were therefore carried out at 275 nm.

### 2.2. Method Validation

#### 2.2.1. Specificity

Caffeine and its metabolites paraxanthine, theobromine, and theophylline were detected at R_F_ values of 0.25, 0.11, 0.15, and 0.19, respectively (Figure 2a). When saliva was spiked with caffeine (Figure 2b), its R_F_ value was maintained, and a clear separation from saliva components (R_F_ 0.004) was achieved, demonstrating the suitability of the developed chromatographic system for the analysis of salivary caffeine levels.

#### 2.2.2. Linearity

Linearity was established by the construction of calibration curves at a concentration range of 20 to 100 ng/band using three replicate experiments. The coefficients of determination (*R*^2^) were found to be greater than 0.99, demonstrating adequate linearity within the specified concentration range (Table 1).

#### 2.2.3. Sensitivity

The limit of detection (LOD) and limit of quantification (LOQ) for caffeine were calculated from three standard curves by using average slope values and y-intercepts obtained from linear regression analysis. The LOD of caffeine was found to be 2.42 ng/band, and the LOQ was 7.34 ng/band (Table 1), which were similar to or lower than detection and quantification levels established in previous studies [19,21]. Reflecting the variability within the three calibration curve replicates, the 95% confidence intervals (CI) were found to be 0.91–6.44 for LOD and 2.75–19.5 for LOQ.

#### 2.2.4. Accuracy and Precision

The accuracy of the developed HPTLC method was evaluated based on sample recovery. The sample recovery of caffeine was calculated using the percent mean recovery following a standard addition method. The mean percent recoveries of replicate analyses were found to be between 101.06% and 102.50% (Table 2). Percent recovery for inter-day precision ranged between 96.63% and 99.43% and for intra-day precision between 99.21% and 104.37% (Table 3). Moreover, the %RSD values for inter-day and intra-day precision were 0.65–2.74% and 0.97–2.23%, respectively, demonstrating a high degree of precision of the developed method (Table 3). With these results the accuracy and precision of the developed method were within the acceptable limits of the International Council for Harmonisation (ICH) guidelines [23].

#### 2.2.5. Repeatability

The repeatability of the developed HPTLC method was assessed using SD and %RSD. A finding of 1.82% for %RSD was within the acceptable limit set by the ICH guidelines (Table 4), confirming the method’s repeatability with a high level of confidence [23].

#### 2.2.6. Robustness

Small changes to three analysis parameters, mobile phase volume, saturation time, and the mobile phase composition, were made intentionally to evaluate the robustness of the method. Caffeine samples at 50 ng/band were analysed under the specified conditions, yielding accuracy values between 98.62% and 104.42%. The changes were also found to have minimal impacts on caffeine’s R_F_ value (Table 5), demonstrating the robustness of the method.

### 2.3. Optimisation of Saliva Sample Preparation

Centrifuged (CS) and non-centrifuged (NC) saliva samples were evaluated using two distinct dilution methods, 1:1 and 1:0.5 (*v*/*v*) with methanol. This study considered two caffeine saliva levels, 20 ng and 40 ng, and tested four treatment conditions for each: centrifuged 1:1, centrifuged 1:0.5, non-centrifuged 1:1, and non-centrifuged 1:0.5. The mean caffeine recoveries ranged from 19.73 to 20.19 ng/band for the 20 ng samples and 38.97 to 42.04 ng/band for the 40 ng samples (Table 6). A three-way ANOVA analysis (centrifugation × ratio × amount) was performed using the recovery data. The findings indicated that there is a statistically significant (*p* = 0.0164) difference in extraction efficiency (recovery) between the two dilution ratios (1:1 vs. 1:0.5). Post hoc (Tukey) results showed that the mean recovery at 1:0.5 was about 3.7% lower than at 1:1. However, centrifugation (NC vs. CS) and caffeine concentrations (20 vs. 40) had no statistically significant impact, suggesting that neither factor alone caused a measurable difference in recovery. None of the two-way interactions (centrifugation × ratio, centrifugation × amount, ratio × amount) nor the three-way interaction was significant. Thus, there was no evidence that the effect of one factor was dependent on the level of another.

Aiming for maximum recovery, the clinical saliva samples investigated with the developed HPTLC method were, therefore, diluted 1:1 with methanol before analysis. This is a significant simplification of the method compared to a previous study, where saliva was first diluted 1:1 with distilled water, followed by several extraction steps with chloroform and overnight evaporation prior to HPTLC analysis [19].

### 2.4. Application of the Developed HPTLC Method to the Analysis of Caffeine in Saliva Samples

**Figure 3:**
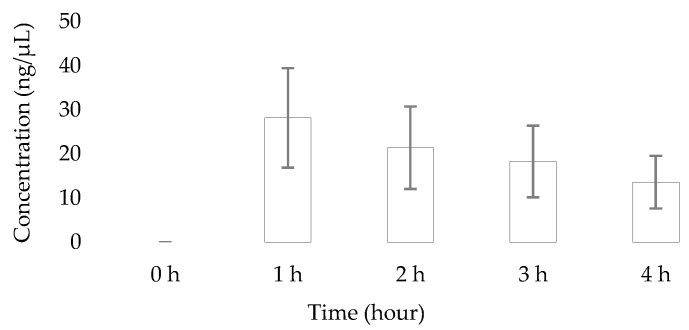
Mean caffeine levels of the investigated clinical samples at different time points.

To demonstrate the applicability of the developed and validated HPTLC method, a total of five saliva sample sets were analysed following a 1:1 (*v*/*v*) dilution with methanol. Each set consisted of five samples (baseline, 1 h, 2 h, 3 h, and 4 h post 100 mg caffeine intake). Caffeine concentrations were mostly found to have peaked around 1 h after ingestion and were lowest at 4 h (Figure 3), which is reflective of trends in salivary caffeine levels reported in previous studies employing either a validated TLC densitometric protocol or differential pulse voltammetry with a glassy carbon electrode for the quantification [16,19]. These similarities serve as further confirmation that the developed HTLC-based approach can be used to monitor salivary caffeine levels for CYP1A2 phenotyping studies.

## 3. Materials and Methods

### 3.1. Chemicals and Reagents

In this study, all chemicals and reagents used were of analytical grade. Caffeine, theophylline, theobromine, paraxanthine, acetone, and chloroform were sourced from Sigma (Sydney, NSW, Australia); methanol was obtained from Scharlau (Barcelona, Spain); and toluene from Honeywell (Muskegon, MI, USA). Moreover, 100 mg No-Doz^®^ caffeine tablets were obtained from Key Pharmaceuticals (Macquarie Park, NSW, Australia). Silica gel 60 F254 HPTLC glass plates (20 cm × 10 cm) were purchased from Merck KGaA (Darmstadt, Germany).

### 3.2. Standard Solution and Mobile Phase Preparation

In this study, 10 mg each of caffeine, theophylline, theobromine, and paraxanthine were dissolved in 10 mL methanol with sonication to prepare the respective stock solutions (1000 ng/µL). For validation of the method, a stock solution of caffeine at a concentration of 100 ng/µL was prepared by dissolving 10 mg of caffeine in 100 mL of methanol, and then from that stock solution, 6 ng/µL and 10 ng/µL working solutions were prepared by further dilution with methanol. Additionally, 20 ng/µL and 5 ng/µL caffeine solutions were prepared from the stock solution via dilution in methanol for spiked saliva analysis. All sample solutions were stored at 4 °C and used for further analysis after being restored to room temperature.

### 3.3. HPTLC Instruments and Method Development

#### 3.3.1. Standard and Sample Application

A semi-automated HPTLC applicator (Linomat 5, CAMAG, Muttenz, Switzerland) was utilised to apply the standard and sample solutions onto the HPTLC plates. For the preparation of the calibration curve, application volumes of the 10 ng/µL caffeine standard ranged from 2 to 10 µL; for analysing recovery, application volumes of the 6 ng/µL caffeine standard ranged from 4 to 6 µL and from 4 to 15 μL for saliva samples. The standard solutions were applied 8 mm from the bottom edge and 20 mm from the side edge of the HPTLC plate at a rate of 80 nLs^−1^ and the saliva samples at 50 nLs^−1^ by using nitrogen gas at 600 kPa, resulting in 8.0 mm long bands that were spaced 11.4 mm apart. A concentration range of 20–100 ng/μL was used to construct the calibration curve for caffeine quantification.

#### 3.3.2. Sample Development

Silica gel 60 F254 HPTLC plates (glass plates 20 × 10 cm^2^) were used as the stationary phase for the chromatographic separation of caffeine. HPTLC plates were developed using acetone/toluene/chloroform (4:3:3, *v*/*v*/*v*) as the mobile phase at ambient temperature in an activated (33% relative humidity) and saturated automated development chamber (ADC2, CAMAG). After 20 min of chamber saturation with the selected mobile phase using a saturation pad, the plates were pre-conditioned with the mobile phase for 5 min before being developed to a migration distance of 80 mm. After development, the plates were dried for 5 min before the plate images were captured using the HPTLC imaging device TLC Visualiser 2 (CAMAG) at 254 nm. The plates were then scanned at 275 nm in absorbance mode using the TLC Scanner 4 (CAMAG) using a scanning speed of 20 mms^−1^, data resolution of 100 µm/step, and slit dimensions of 5 × 0.2 mm^2^. The HPTLC software (VisionCATS 4.0, CAMAG) was used to operate the instrument modules and analyse all generated data.

### 3.4. Method Validation

The developed HPTLC method was validated for specificity, linearity, sensitivity, precision, accuracy, repeatability, and robustness according to the International Conference on Harmonisation (ICH) guidelines Q2 (R2) [23].

#### 3.4.1. Specificity

Specificity refers to the capability of the method to detect the analyte of interest in the presence of interferents, degradation products, or other compounds in the matrix. By using HPTLC, specificity is assured by a distinct peak and retention factor (R_F_) of the analyte compared to other compounds in the matrix. To evaluate specificity, caffeine and its metabolites paraxanthine, theophylline, and theobromine were analysed as separate samples and as an over-spotted mixture where the concentration of all the samples was 1000 ng/band. For the confirmation that caffeine was also separated from any inherent saliva constituents, the R_F_ values of detected bands in 10 µL blank saliva (diluted 1:1 with methanol) were compared to that of the caffeine standard (500 ng/band) and saliva samples diluted 1:1 with methanolic caffeine solution (equivalent to 500 ng/band caffeine).

#### 3.4.2. Linearity

Linearity refers to the method’s ability to generate proportional results for the analyte’s peak height over a range of concentrations. In this study, five-point calibration curves with caffeine amounts varying from 20 ng/band to 100 ng/band were used to evaluate the linearity and range of the developed method. All analyses were performed in triplicate, and linearity was determined by the coefficient of determination (*R*^2^), slope (m), y-intercept, and standard deviation (SD) of the line of best fit of the calibration curves. Microsoft Excel was used for the calculations.

#### 3.4.3. Sensitivity

Sensitivity is defined as the ability of the method to identify and quantify small concentrations of analyte in a sample matrix and is expressed as the limit of detection (LOD) and limit of quantification (LOQ). To determine LOD and LOQ, calibration curves were prepared from 5 different caffeine amounts ranging from 20 to 100 ng/band. LOD and LOQ values were then calculated using the following equations as stipulated in the ICH guidelines [23]:

LOD = 3.3 σ/S(1)

and

LOQ = 10 σ/S (2)

where σ is the mean standard deviation (SD) of the y-intercept and S is the average slope value of triplicate calibration curves.

#### 3.4.4. Accuracy

The accuracy of the method refers to the ability to produce results that are close to the expected value of the samples being analysed. To determine the accuracy of the method, three different amounts of caffeine equivalent to 80%, 100%, and 120% of the target amount (30 ng/band) were quantified. The analysis was performed in triplicate with accuracy reported as the % recovery and %RSD of the analysed caffeine standards.

#### 3.4.5. Precision

Precision is defined as the ability of the method to produce consistent results with little variation between samples that are analysed within the same prescribed conditions but at different times on the same day (i.e., intra-day precision) and on different days (i.e., inter-day precision), in different laboratories, and/or by different instruments. In this study, intra-day and inter-day precision were determined using three different amounts of caffeine (24, 30, and 36 ng/band) analysed in triplicate. For intra-day precision, the analysis was performed three times on the same day and for inter-day precision once on three different days, with precision then being expressed as percent relative standard deviation (%RSD) and percent recovery of the results obtained.

#### 3.4.6. Repeatability (System Precision)

The ability to obtain precise results under consistent operating conditions within a short time frame demonstrates the repeatability of an analytical method. The repeatability of this HPTLC method was confirmed by analysing a standard caffeine solution at an amount of 30 ng/band five times and expressed as %RSD.

#### 3.4.7. Robustness

Three parameters were studied to evaluate the robustness of the method for analysing caffeine: a slight change in the mobile phase volume (±2 mL), saturation time of the development chamber (±5 min), and mobile phase composition (acetone/toluene/chloroform (4:4:3 and 4:3:4 *v*/*v*/*v*). In all robustness experiments, the respective effect on recovery and the R_F_ value for caffeine were determined. The theoretical amount of caffeine in these experiments was 50 ng/band.

### 3.5. Method Optimisation for Saliva Sample Preparation

In this study, some modifications to a previously reported saliva sample preparation were made in order to optimise the analysis and improve its efficiency [19]. To assess the best way to prepare saliva for analysis, centrifuged and non-centrifuged saliva samples were prepared. Blank saliva was collected from a volunteer who had abstained from caffeine intake for 8 h prior. Furthermore, 1 mL of saliva was transferred into 1.5 mL Eppendorf tubes using a plastic syringe. Samples that required centrifugation were centrifuged (Centrifuge MiniSpin, Australia) at 7.50 × 1000 rpm for 15 min, and the resulting supernatant was used for spiking with caffeine. All saliva samples except the blank saliva samples were diluted either 1:1 or 1:0.5 (*v*/*v*) with methanolic caffeine solution (20 ng µL^−1^ or 5 ng µL^−1^). Blank saliva samples were prepared by diluting 1:1 or 1:0:5 (*v*/*v*) with methanol only. All Eppendorf tubes were vortexed for 15 s prior to application of the samples onto the HPTLC plate (20 ng/band and 40 ng/band). Before each HPTLC run, a total of 8 samples was prepared, and their caffeine content was determined using a five-point calibration curve (20 to 100 ng/band). The analysis was performed in replicate, and accuracy and precision were reported as %recovery and %RSD values, respectively. Three-way ANOVA (centrifugation × ratio × amount) analysis was used to optimise saliva preparation technique based on the % recovery of caffeine in saliva.

### 3.6. Human Ethics Approval and Clinical Saliva Sample Collection

The novel assay was applied to quantify caffeine levels in the saliva of participants who had taken caffeine tablets via the oral route. This study was approved by the Human Research Ethics Committee of The University of Western Australia (Reference No.: 2024/ET000285). Third-year pharmacology students at the University of Western Australia (UWA) were invited to participate in this study. Before ingestion of caffeine, subjects first completed a Pretreatment Participant Questionnaire (PPQ) seeking information on prior adverse experiences with caffeine as well as their use of CYP1A2-modifying pharmaceuticals. Those reporting headaches and other signs of caffeine intolerance, a history of phenylketonuria, currently taking strong CYP1A2 inhibitor medicines (e.g., fluvoxamine or ciprofloxacin), and/or currently using strong CYP1A2 inducers (e.g., carbamazepine) were excluded from participation in this study. Following this protocol, a total of 23 students were recruited, and the collated data of five of them were used to demonstrate the applicability of the developed HPTLC method for CYP1A2 phenotyping using caffeine as a probe drug. After the participants had provided written informed consent, they were asked to abstain from caffeine intake and to fast for at least 4 h on the day of the trial. They were provided with sugarless chewing gum (Wrigley’s Extra) to stimulate salivation as required, and 7 mL of saliva was collected from each participant for baseline measurement. Each participant then swallowed two 100 mg No-Doz^®^ caffeine tablets with 250 mL of water and provided 3-millilitre saliva samples after chewing sugarless chewing gum to assist saliva production at 1 h, 2 h, 3 h, and 4 h post-administration. Saliva was collected into a 50 mL centrifuge tube with a screw cap and conical bottom. A total of 25 saliva samples were collected from the 5 participants (5 samples per participant). The saliva samples were immediately stored at −20 °C until HPTLC analysis. Caffeine content in the saliva samples were analysed using the validated HPTLC method to demonstrate its value for potential future CYP1A2 phenotyping studies.

## 4. Conclusions

This study successfully developed and validated a HPTLC method for detecting and quantifying caffeine in methanolic solutions with a LOD and LOQ of 2.42 and 7.34 ng/band, respectively. An optimised 1:1 saliva/methanol sample dilution process was used to prepare spiked saliva samples as well as to process clinical samples before the HPTLC analysis. The HPTLC method was successfully applied to the analysis of salivary caffeine levels in five participants at different time points (0, 1, 2, 3, and 4 h) following caffeine consumption, demonstrating its application in routine caffeine analysis in human saliva samples. This study demonstrates that HPTLC analysis can produce quantitative data in support of CYP1A2 phenotype assessments without the need for extensive sample preparation. It is therefore an efficient and cost-effective alternative to other analytical methods (e.g., HPLC or ELISA) employed for this purpose.

## Figures and Tables

**Table 1:** Linear regression analysis for the calibration curves of caffeine.

R_F_	Run	Linearity Range (ng/Band)	Regression Equation	Correlation Coefficient (*R*^2^)	Slope (m)	Slope (Average)	y-Intercept	SD of y-Intercept	LOD (ng/Band)	LOQ (ng/Band)	95% CI (ng/Band)
0.25	Run 1	20–100	y = 1 × 10^−3^x + 3.9 × 10^−3^	0.9990	9.57 × 10^−4^	1.01 × 10^−3^	5.04 × 10^−3^	7.41 × 10^−4^	2.42	7.34	LOD: 0.91–6.44 LOQ: 2.75–19.5
	Run 2	20–100	y = 1 × 10^−3^x + 4.9 × 10^−3^	0.9970	1.042 × 10^−3^		4.92 × 10^−3^				
	Run 3	20–100	y = 1 × 10^−3^x + 6.3 × 10^−3^	0.9964	1.032 × 10^−3^		6.26 × 10^−3^				

**Table 2:** Recovery of caffeine.

Theoretical Amount (ng/Band)	Run 1			Run 2			Run 3		
	Amount Recovered (ng/Band)	% Recovery	% Mean Recovery	Amount Recovered (ng/Band)	% Recovery	% Mean Recovery	Amount Recovered (ng/Band)	% Recovery	% Mean Recovery
24	24.81	103.38		24.04	100.17		24.42	101.75	
30	31.68	105.60	102.50	30.94	103.13	101.06	31.48	104.93	101.99
36	35.47	98.53		35.96	99.89		35.74	99.28	

**Table 3:** Inter-day and Intra-day precision (n = 3).

Theoretical Amount (ng/Band)	Inter-Day			Intra-Day		
	Amount Recovered (ng/Band) (Mean ± SD)	%RSD	% Recovery	Amount Recovered (ng/Band) (Mean ± SD)	%RSD	% Recovery
24	23.19 ± 0.15	0.65	96.63	24.43 ± 0.54	2.23	101.77
30	29.77 ± 0.92	3.08	99.23	31.31 ± 0.52	1.67	104.37
36	35.80 ± 0.98	2.74	99.43	35.72 ± 0.35	0.97	99.21

**Table 4:** Repeatability (system precision).

Caffeine Theoretical Amount (ng/Band)	Caffeine Measured Amount (ng/Band)
30	29.73
30	30.89
30	31.09
30	30.86
30	31.01
Average	30.72
SD	0.56
%RSD	1.82

**Table 5:** Robustness (changes in different parameters).

Parameters	Theoretical Amount (ng/Band)	% Recovery	R_F_ Value (Mean ± SD)
**Mobile Phase Development Amount (mL)**			
8	50	104.42	0.25 ± 0.01
12	50	102.18	0.25 ± 0.03
**Saturation Time (minutes)**			
15	50	100.60	0.25 ± 0.01
25	50	98.88	0.25 ± 0.02
**Mobile Phase Composition**			
Acetone/Toluene/Chloroform (4:4:3)	50	98.66	0.25 ± 0.01
Acetone/Toluene/Chloroform (4:3:4)	50	99.62	0.25 ± 0.03

**Table 6:** Recovery of caffeine in spiked saliva samples using four different treatment methods.

Parameters	Theoretical Amount (ng/Band)	Recovered Amount (ng/Band)			Mean	SD	%RSD
		Run 1	Run 2	Run 3			
Non-centrifuged (1:1)	20	19.75	20.61	20.21	20.19	0.43	2.15
Centrifuged (1:1)	20	19.13	21.04	19.97	20.05	0.96	4.78
Non-centrifuged (1:0.5)	20	18.93	19.89	20.23	19.68	0.67	3.43
Centrifuged (1:0.5)	20	19.49	19.44	20.25	19.73	0.45	2.30
Non-centrifuged (1:1)	40	40.14	41.39	41.99	41.17	0.94	2.29
Centrifuged (1:1)	40	41.67	42.64	41.82	42.04	0.52	1.24
Non-centrifuged (1:0.5)	40	37.68	41.60	40.62	39.97	2.04	5.10
Centrifuged (1:0.5)	40	40.42	39.10	37.40	38.97	1.51	3.88

## Data Availability

The original contributions included in this study are presented within this article. Further inquiries can be sent to the corresponding author.

## Supplementary Materials

The following supporting information can be downloaded at: https://www.mdpi.com/article/10.3390/molecules30193859/s1. Figure S1: Absorption spectra of caffeine in methanol (blue line) as well as caffeine in spiked saliva (red line) obtained by CAMAG TLC Scanner 4.
