# Supplementary material for: Development of a Validated High-Performance Thin-Layer Chromatography (HPTLC) Analysis Protocol for Salivary Caffeine Used as a Probe Drug
Source: Molecules. 2025 Sep 23;30(19):3859. doi: 10.3390/molecules30193859 (PMC12525945; doi:10.3390/molecules30193859)
Supplement: Supplementary file 1 [file molecules-30-03859-s001.zip › molecules-3867042-supplementary.pdf]

Supplementary Material

Sikdar et al. (2025) "Development of a validated High-Performance Thin-Layer Chromatography (HPTLC) analysis protocol for salivary caffeine used as a probe drug"

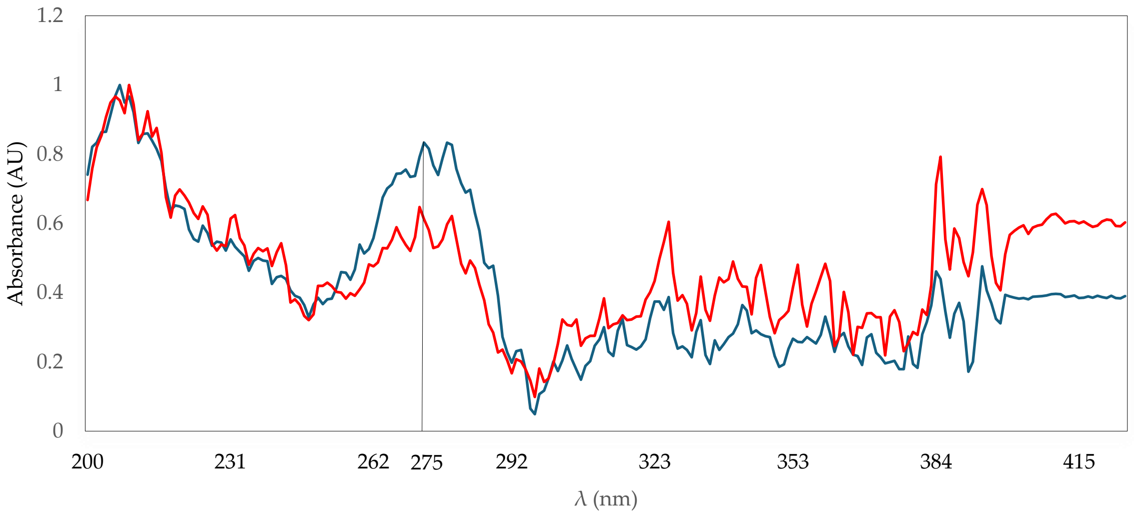

Figure S1. Absorption spectra of caffeine in methanol (blue line) as well as caffeine in spiked saliva (red line) obtained by CAMAG TLC Scanner 4.
